# Hemizygous deletion of *CDKN2A/B* in *IDH*-mutated glioma: Prognostic impact when adjusting for clinical factors

**DOI:** 10.1093/noajnl/vdag096

**Published:** 2026-04-13

**Authors:** Anna Lipatnikova, Franziska M Ippen, Anna Dénes, Louise Carstam, Thomas Olsson Bontell, Alba Corell, Sandra Ferreyra Vega, Ryan Shun-Yuen Kwan, Abigail K Suwala, Helena Carén, Asgeir S Jakola

**Affiliations:** Department of Clinical Neuroscience, Institute of Neuroscience and Physiology, Sahlgrenska Academy, University of Gothenburg, Gothenburg, Sweden; National Center for Tumor Diseases (NCT), NCT Heidelberg, A Partnership Between DKFZ and University Hospital Heidelberg, Heidelberg, Germany; Clinical Cooperation Unit Neuropathology, German Cancer Research Center (DKFZ), German Consortium for Translational Cancer Research (DKTK), Heidelberg, Germany; Department of Neurology, University Hospital Heidelberg, Heidelberg, Germany; Department of Clinical Neuroscience, Institute of Neuroscience and Physiology, Sahlgrenska Academy, University of Gothenburg, Gothenburg, Sweden; Department of Clinical Neuroscience, Institute of Neuroscience and Physiology, Sahlgrenska Academy, University of Gothenburg, Gothenburg, Sweden; Department of Neurosurgery, Sahlgrenska University Hospital, Gothenburg, Sweden; Department of Physiology, Institute of Neuroscience and Physiology, Sahlgrenska Academy, University of Gothenburg, Gothenburg, Sweden; Department of Clinical Pathology, Sahlgrenska University Hospital, Gothenburg, Sweden; Department of Clinical Neuroscience, Institute of Neuroscience and Physiology, Sahlgrenska Academy, University of Gothenburg, Gothenburg, Sweden; Department of Neurosurgery, Sahlgrenska University Hospital, Gothenburg, Sweden; Sahlgrenska Center for Cancer Research, Department of Medical Biochemistry and Cell Biology, Institute of Biomedicine, Sahlgrenska Academy, University of Gothenburg, Gothenburg, Sweden; Sahlgrenska Center for Cancer Research, Department of Medical Biochemistry and Cell Biology, Institute of Biomedicine, Sahlgrenska Academy, University of Gothenburg, Gothenburg, Sweden; Clinical Cooperation Unit Neuropathology, German Cancer Research Center (DKFZ), German Consortium for Translational Cancer Research (DKTK), Heidelberg, Germany; Department of Neuropathology, University Hospital Heidelberg, Heidelberg, Germany; Sahlgrenska Center for Cancer Research, Department of Medical Biochemistry and Cell Biology, Institute of Biomedicine, Sahlgrenska Academy, University of Gothenburg, Gothenburg, Sweden; Department of Clinical Neuroscience, Institute of Neuroscience and Physiology, Sahlgrenska Academy, University of Gothenburg, Gothenburg, Sweden; Department of Neurosurgery, Sahlgrenska University Hospital, Gothenburg, Sweden

**Keywords:** astrocytoma, *CDKN2A/B*, low-grade glioma, survival

## Abstract

**Background:**

There is inconsistent evidence if hemizygous *CDKN2A/B* deletion affects survival in *IDH*-mutated glioma, and clinical characteristics are rarely accounted for. This study aims to investigate overall survival and clinical characteristics of *IDH*-mutated glioma with a hemizygous deletion of *CDKN2A/B*.

**Method:**

A total of 215 consecutive patients with *IDH*-mutated glioma, WHO grade 2 and 3, who underwent primary surgery between 2007 and 2023 in a defined catchment area were included. *CDKN2A/B* status was determined through visual assessment of DNA methylation array-derived copy-number plots. Multivariable analyses and propensity score matching were performed to consider other clinical factors.

**Results:**

A *CDKN2A/B* hemizygous deletion was identified in 24/215 patients, including 22/116 with astrocytoma and 2/99 with oligodendroglioma. Due to this imbalance across subtypes, we primarily focused on astrocytomas. Hemizygous deletion was present in 9/65 patients with WHO grade 2 and 13/51 with WHO grade 3 astrocytomas. Patients with astrocytoma and hemizygous *CDKN2A/B* deletion had shorter overall survival compared to patients with intact *CDKN2A/B* status (5.8 years vs 11.4 years, *P* = .04). *CDKN2A/B* was a survival predictor in WHO grade 2 astrocytoma but not in WHO grade 3. However, these effects disappeared both in the multivariable analysis and in the propensity-matched cohort.

**Conclusion:**

The presence of a hemizygous *CDKN2A/B* deletion occurs more frequently in astrocytomas compared to oligodendrogliomas at the time of primary diagnosis. Worse survival in patients with astrocytoma and *CDKN2A/B* hemizygous loss was observed, specifically in WHO grade 2, but this prognostic effect disappeared when adjusting for clinical factors.

## Graphical Abstract

**Graphical Abstract:**
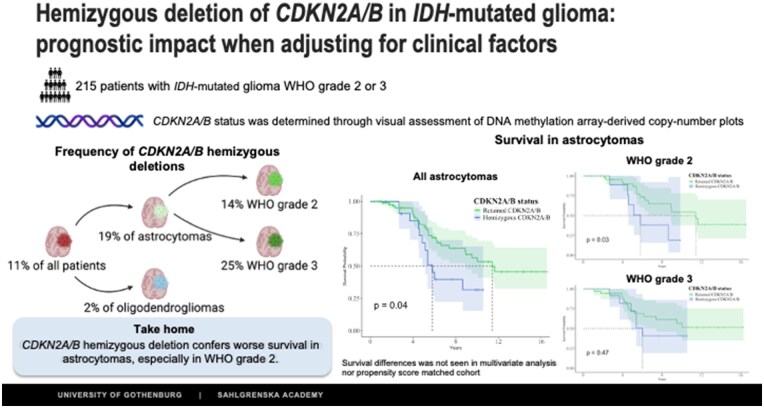

Key Points

- A *CDKN2A/B* hemizygous deletion was prognostic in *IDH*-mutated astrocytomas.
- In WHO grade 2 astrocytomas, *CDKN2A/B* hemizygous loss was particularly impactful.
- After adjustment for clinical factors, only tumor burden remained a significant prognostic factor.

Importance of the Study

There is increasing interest in *CDKN2A/B* hemizygous deletion as a prognostic marker for poor prognosis in *IDH*-mutant gliomas, but the impact of *CDKN2A/B* hemizygous deletion remains uncertain. Key studies using different methods have reached different conclusions. Here, we use a described and easily accessible method to characterize *CDKN2A/B* hemizygous deletions. We demonstrate that *CDKN2A/B* hemizygous deletion is prognostic, with particular impact in WHO grade 2 astrocytomas. Still, after adjusting for known clinical prognostic factors, the prognostic importance diminished.

In the latest WHO classification of central nervous system tumors, molecular markers have gained increased relevance for both diagnostic and grading purposes. Among key additions is the incorporation of *CDKN2A/B* homozygous deletions (HomDel) in the grading system for *IDH*-mutated astrocytomas. A *CDKN2A/B* HomDel is a criterion for classifying an *IDH*-mutated astrocytoma as WHO grade 4.^1–3^ The distinction between *IDH*-mutated astrocytoma WHO grades 2 and 3 relies solely on histopathological features.^3^

The prognostic significance of *CDKN2A/B* hemizygous deletion (HemiDel) in gliomas has received increasing attention. In astrocytomas, some studies have demonstrated shorter survival in tumors harboring a *CDKN2A/B* HemiDel,^4–7^ while others have reported no difference between patients with HemiDel and intact *CDKN2A/B* status.^8–10^ In oligodendrogliomas, alterations in *CDKN2A/B* are less frequent.^5^ Several studies have associated *CDKN2A/B* HomDel with shorter survival in oligodendrogliomas,^1^^,^^8^^,^^11^ but the prognostic significance remains controversial.^12^^,^^13^  *CDKN2A/B* HemiDel is less investigated in oligodendroglioma, but available data suggest no prognostic impact of HemiDel compared with retained status.^8^^,^^11^

A shortcoming of previous studies is that they have examined the relationship between *CDKN2A/B* HemiDel and survival without adjusting for established clinical variables known to influence clinical decision making, such as age, tumor burden, and WHO grade.^4^^,^^8^

In this population-based study, we aimed to study the prognostic impact of *CDKN2A/B* HemiDel in *IDH*-mutated astrocytomas and oligodendrogliomas WHO grades 2 and 3 while adjusting for clinical factors. We hypothesized that *CDKN2A/B* HemiDel influences survival in both glioma subtypes.

## Method

### Study Design

This retrospective population-based study defined the following inclusion criteria: (1) underwent surgery at Sahlgrenska University hospital (Gothenburg, Sweden) between 2007 and 2023, with either biopsy or resection; (2) age 18 years or above; and (3) diagnosis with either an *IDH*-mutated astrocytoma or oligodendroglioma WHO grade 2 or 3 according to the 2021 WHO classification system as primary diagnosis.^3^

Exclusion criteria were: (1) another concurrent malignancy; (2) a previous diagnosis of another primary brain tumor; and (3) unavailable tissue for DNA extraction.

### Study Population

Clinical and pathological data were collected retrospectively from medical records. End of follow-up was set to January 1, 2024.

Tumor volume was assessed by using 3D slicer (version 5.6.2). T2-weighted images (T2 or FLAIR sequences) were used in all cases except 3 where T1-weighted post contrast (T1Gd) was used. Postoperative tumor volume was divided into RANO classes: where class 1 is complete resection with no residual tumor volume; class 2, near total resection with <5 cm^3^ residual tumor volume; class 3, subtotal resection with residual tumor volume ranging from 5 to 25 cm^3^; and class 4, partial resection with >25 cm^3^ residual tumor volume.^14^ All segmentations were reviewed by an experienced neurosurgeon.

### Molecular Assessment

DNA was extracted from formalin-fixed paraffin-embedded or fresh-frozen tissue. Subsequently, generation of genome-wide DNA methylation array data was performed as described in previous publications.^15^ Chromosomal copy number alteration plots were generated from raw DNA methylation array data (Illumina methylationEPIC array) using the package conumee 2.0 in R software and R studio (version 4.0.2).^16^ The plots were visually assessed by 2 independent evaluators with methods described by Ippen et al.^8^ In short, the *CDKN2A/B* locus was assessed in relation to larger segmental losses (and gains). If the locus was at the level of a chromosomal loss, it was classified as a HemiDel. When the signal was approximately twice the distance of a chromosomal loss, or larger segmental loss, the alteration was interpreted as HomDel. In the absence of larger chromosomal losses, the locus was assessed by comparison with the baseline and smaller focal losses.

### Statistics

All statistics were performed in R using R Studio (version 2025.05.0 + 496). Binary variables were compared using Fisher’s exact test, while continuous variables were analyzed with Wilcoxon rank sum test since data deviated from normal distribution. Survival was estimated using the Kaplan–Meier method, and differences between groups were assessed with the log-rank test within the survival package.^17^ Multivariable Cox proportional hazard models were constructed for astrocytoma (using survival package) to control for factors that has been associated with survival, such as age,^18^^,^^19^ histopathological grade,^3^^,^^20^ postoperative tumor volume,^21^^,^^22^ and oncological treatment.^23^ Statistical significance was defined as *P* < .05. Patients with astrocytoma harboring a *CDKN2A/B* HemiDel were matched 1:1 to patients without HemiDel through nearest neighbor propensity score matching, implemented with the MatchIt package.^24^ Matching was performed on patient’s age at diagnosis, histopathological grade, preoperative tumor volume, and postoperative tumor volume. Descriptive and survival analyses in the matched cohort were performed according to the statistical methods described above.

## Results

**Figure 1.**
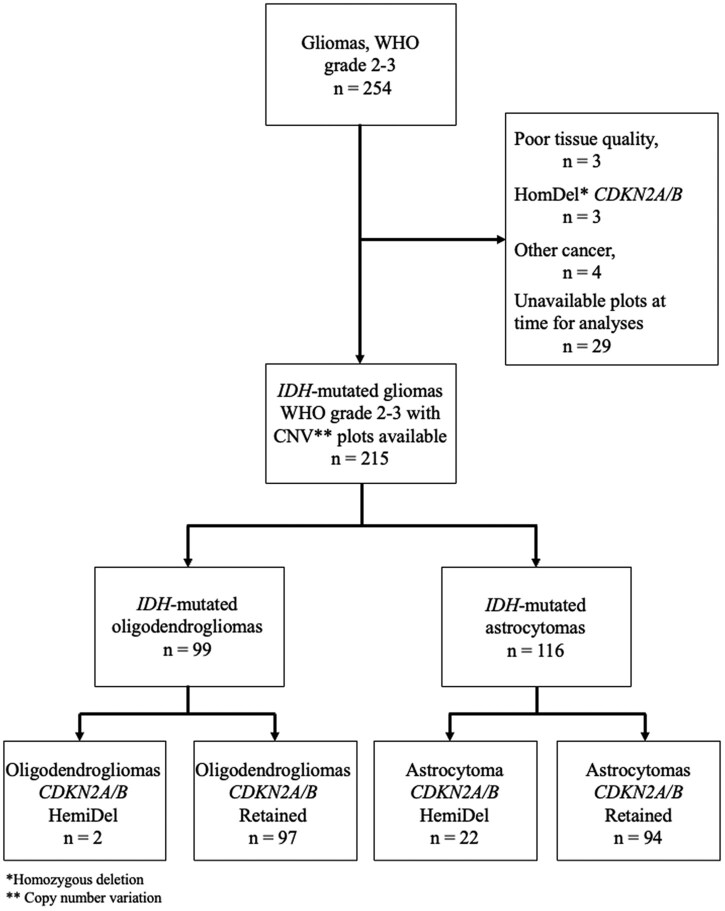
Flow chart of patients included in this study.

A total of 215 patients were included in this study (see Figure 1). Of these, 116 had astrocytomas, comprising 65 (56%) WHO grade 2 and 51 (44%) WHO grade 3 astrocytomas. The remaining 99 patients had oligodendroglioma, including 48 (48%) WHO grade 2 and 51 (51%) WHO grade 3 oligodendroglioma. Overall, 24 (11%) patients had tumors harboring a HemiDel *CDKN2A/B*, and the remaining 191 (91%) had retained *CDKN2A/B* status. The proportion of tumors with *CDKN2A/B* HemiDel was higher in astrocytomas than in oligodendrogliomas (19% compared with 2%, *P* < .001). As only 2 patients with oligodendroglioma had *CDKN2A/B* HemiDel, the analysis presented below focuses on astrocytomas. We did not observe any significant differences in baseline or treatment characteristics between astrocytomas harboring a *CDKN2A/B* HemiDel and those with retained status. For the important prognostic factor of preoperative tumor burden, patients with HemiDel had a median tumor volume of 73 cm^3^ compared with 52 cm^3^ in patients with retained *CDKN2A/B* status (*P* = .06, Table 1). Baseline characteristics for the full cohort, including the oligodendrogliomas, are presented in Table S1.

**Table 1.** Baseline and treatment characteristics of *IDH*-mutated astrocytomas, WHO grades 2 and 3 with retained or hemizygous *CDKN2A/B* deletion status

	HemiDel, *n* = 22	Retained, *n* = 94	*P* value
Male, *n* (%)	15 (68)	54 (55)	.47
Age, median (Q1, Q3)	37 (32, 50)	35 (29, 45)	.16
Tumor grade, *n* (%)			.15
Grade 2	9 (41)	56 (60)	
Grade 3	13 (59)	38 (40)	
Symptoms at diagnosis, *n* (%)^a^			
Seizures	15 (68)	72 (77)	.42
Headache	7 (32)	17 (18)	.16
Motor deficit	3 (17)	9 (10)	.70
Language deficit	3 (17)	10 (11)	.71
Visual deficit	1 (5)	2 (2)	.47
Tumor location, *n* (%)			.24
Frontal	9 (41)	44 (47)	
Temporal	4 (18)	28 (30)	
Other	9 (41)	22 (23)	
Bilateral extension, *n* (%)	3 (17)	6 (6)	.37
Type of surgery, *n* (%)			1.00
Biopsy	0 (0)	3 (3)	
Resection	22 (100)	91 (97)	
Oncological treatment, *n* (%)			.84
Combination treatment^b^	15 (68)	51 (54)	
Chemotherapy or radiotherapy	6 (27)	32 (34)	
No treatment	1 (5)	9 (10)	
Tumor volume preoperatively (ml), median (Q1, Q3)	73 (55, 103)	52 (27, 94)	.06
RANO, *n* (%)^c^			.27
Class 1-2	5 (23)	39 (41)	
Class 3	8 (36)	26 (28)	
Class 4	8 (36)	25 (27)	

a Several symptoms at time of diagnosis are possible, why >100% is possible.

b Radiotherapy with adjuvant chemotherapy or treatment according to the STUPP protocol.

c Numbers do not reach 100% due to missing data.

### Survival

**Figure 2.**
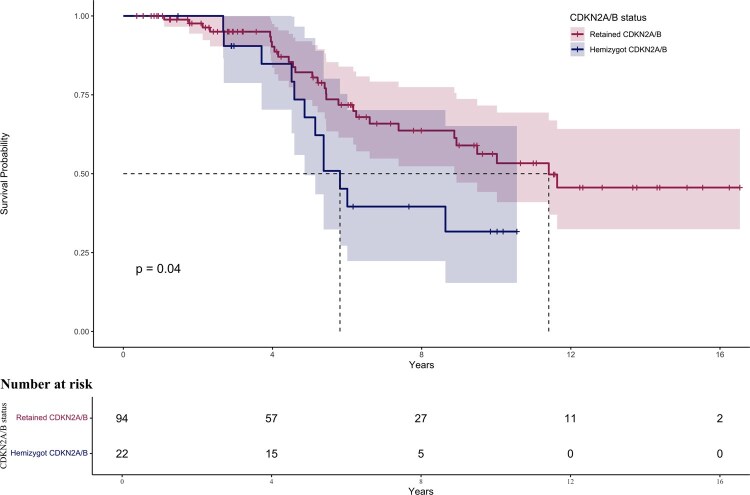
Kaplan–Meier survival curves for astrocytomas stratified by *CDKN2A/B* status. Patients with *CDKN2A/B* HemiDel had significantly shorter survival than those with retained *CDKN2A/B* (log-rank, *P* = 0.04).

The median overall survival for the full cohort was 14.3 years (Q1: 6.2 years, Q3: not reached). In patients with astrocytomas, a significant shorter survival was seen in tumors presenting with *CDKN2A/B* HemiDel compared with retained *CDKN2A/B* status, with a median of 5.8 years (Q1: 4.8 years, Q3: not reached) compared to 11.4 years (Q1: 5.4 years, Q3: not reached, log-rank *P* = .04, Figure 2). For the full cohort and in patients with oligodendrogliomas, see Figure S1A and B.

**Figure 3.**
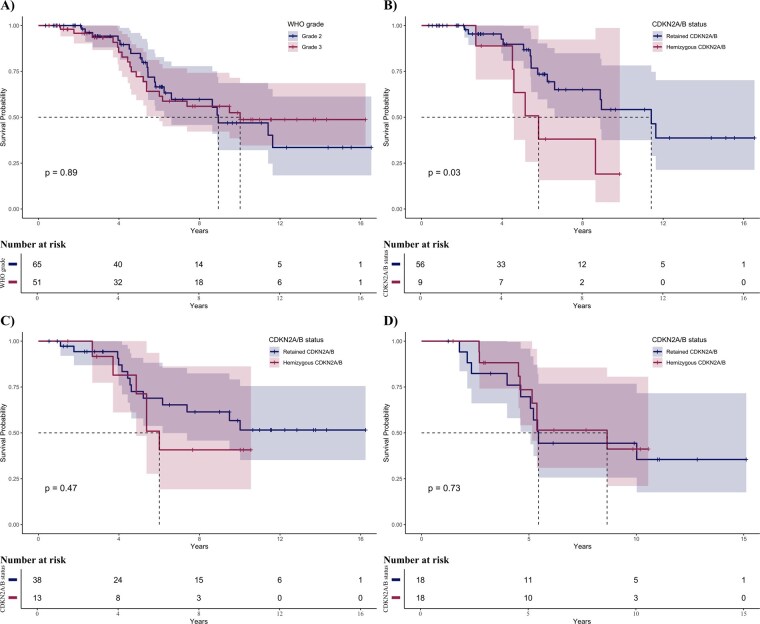
Survival analyses for subgroups of patients with *IDH* mutated astrocytoma, WHO grades 2 and 3. (A) Kaplan–Meier curves stratified by WHO grade. No significant difference on survival was seen (log-rank, *P* = .89). (B) In a subgroup of WHO grade 2 astrocytomas, we stratified by *CDKN2A/B* status. Patients with hemizygous *CDKN2A/B* status had worse survival compared with patients with retained *CDKN2A/B* status (log-rank, *P* = .03). (C) When we stratified WHO grade 3 astrocytomas by *CDKN2A/B* status, we did not observe any survival difference (log-rank, *P* = .47). (D) We analyzed *CDKN2A/B* status in a propensity score-matched cohort. We matched 1:1 with controls considering the patient age, histopathological grade, preoperative tumor volume, and postoperative RANO class, creating overall a well-balanced comparison group. In this cohort, no survival effect based on *CDKN2A/B* was found (log-rank, *P* = .45).

When analyzed by WHO grade, no significant survival difference was found between WHO grades 2 and 3 astrocytomas (log-rank *P* = .89, Figure 3A). When stratified by *CDKN2A/B*, HemiDel was associated with significantly shorter survival in WHO grade 2 astrocytomas (5.8 years compared to not reached in tumors with retained *CDKN2A/B*, log-rank *P* = .03) but not in WHO grade 3 astrocytomas (log-rank *P* = .47, Figure 3B and C).

In a multivariable Cox proportional hazard model of astrocytomas, adjusting for age at primary surgery, histological grade, RANO class, and oncological treatment, *CDKN2A/B* status was not significantly associated with survival. Only RANO class reached statistical significance in this model (*P* < .01, Table 2).

**Table 2.** Results from the multivariable Cox proportional hazard analysis of astrocytomas.

	Astrocytomas	
Variable	HR (95% CI)	*P* value
*CDKN2A/B* Hemidel (vs. retained)	1.53 (0.61-3.87)	.36
WHO grade 3 (vs. WHO grade 2)	0.93 (0.35-2.49)	.89
Age at primary surgery	0.99 (0.95-1.02)	.57
RANO class 3 (vs. RANO class 1–2)	6.00 (1.71-20.80)	**<.01**
RANO class 4 (vs. RANO class 1–2)	7.65 (2.00-29.34)	**<.01**
Combination treatment (vs. nothing)	0.28 (0.03-2.81)	.28
Radiation therapy (vs. nothing)	1.11 (0.12-9.97)	.93
Chemotherapy (vs. nothing)	0.58 (0.05-6.28)	.66

Abbreviations: 95% CI, 95% confidence interval; HR, hazard ratio.

The propensity-matched groups were similar in baseline characteristics (Tables S2 and S3). There were no survival differences in this propensity-matched cohort based on *CDKN2A/B* status (log-rank *P* = .45, Figure 3D).

## Discussion

In our population-based, single-center study, *CDKN2A/B* HemiDel was more commonly observed in astrocytoma than in oligodendroglioma. This difference in frequencies made further analysis of the full cohort inappropriate, as the apparent prognostic effect of *CDKN2A/B* was likely driven by the uneven distribution of the alteration across glioma subtypes. Patients with astrocytomas harboring a *CDKN2A/B* HemiDel had shorter survival compared with a retained *CDKN2A/B* status. This survival difference appears to exceed any potential survival distinction between WHO grades 2 and 3 astrocytomas. However, the prognostic impact of *CDKN2A/B* HemiDel diminished when accounting for other factors in a multivariable model and a propensity-matched cohort.

Previous studies have shown an association between *CDKN2A/B* HemiDel and survival in univariable analysis,^4–7^ but findings are not consistent.^8–10^ One reason for the heterogeneity of findings could be due to the lack of consensus on the optimal method for classifying *CDKN2A/B* alterations, which is why different methods are used across studies.^2^^,^^8^^,^^10^^,^^20^^,^^25^ Among these, DNA-methylation profiling has emerged as one of the most common and robust approaches and the one used in our study. Still, different cutoffs have been used in the assessment of *CDKN2A/*B from DNA methylation profiling.^2^^,^^4^ Such methodological diversity is likely a contributing factor to inconsistent findings regarding the prognostic impact of *CDKN2A/B* HemiDel across different studies.^4–10^

A further consideration is that *CDKN2A/B* HemiDel may not theoretically be expected to act as drivers for worse prognosis, since these are tumor suppressor genes,^26^^,^^27^ which typically require bi-allelic inactivation for full functional loss according to the Knudson hypothesis.^28^ However, a *CDKN2A/B* HemiDel on DNA methylation copy-number plots may be represented by subclonal *CDKN2A/B* HomDel losses, producing a composite signal that is interpreted as HemiDel.^8^^,^^29^ Thus, the prognostic impact of *CDKN2A/B* observed in our cohort may, at least in part, be caused by underlying intratumoral heterogeneity in tumors classified as *CDKN2A/B* HemiDel. Other methods, such as fluorescence in situ hybridization (FISH), can better resolve this ambiguity by counting *CDKN2A/B* signals per nucleus, thus distinguishing between a clonal HemiDel from a subclonal HomDel.^30^ However, FISH is probe- and sampling-dependent and can show reduced concordance, especially in gliomas with higher WHO grade and genomic instability.^31^ An alternative hypothesis is that tumors harboring a *CDKN2A/B* HemiDel could have a higher risk of developing HomDel because of genomic instability, thus driving a more aggressive tumor behavior.

Beyond the survival patterns related to *CDKN2A/B*, our data also provide insights into the underlying tumor biology. The imbalance in preoperative tumor volume, with numerically slightly larger tumors in *CDKN2A/B* HemiDel, may indicate that the genetic alteration occurs at a later stage in the development of *IDH*-mutant astrocytomas. A delayed onset may allow for a period of slower tumor growth, giving the brain time to adapt and postponing the appearance of symptoms. Given that *CDKN2A/B* HomDel is more frequently observed in recurrent tumors compared to primary tumors, strengthen this stepwise malignant evolvement.^15^^,^^25^^,^^32^

The survival disadvantage associated with a *CDKN2A/B* HemiDel was more pronounced in WHO grade 2 astrocytomas. One possible explanation could be a traditionally more aggressive treatment strategy for WHO grade 3 astrocytomas, which could partly mitigate the adverse prognostic effect of *CDKN2A/B* HemiDel, particularly with the intermediate follow-up available. However, in our cohort, overall treatment strategies were comparable across *CDKN2A/B* status. In addition, no statistically significant treatment effect was observed in the multivariable analysis. Therefore, we think that these small and nonsignificant differences in treatment strategy across *CDKN2A/B* status are unlikely to fully account for the observed pattern.

In our multivariable analysis, *CDKN2A/B* HemiDel had no impact on survival, nor was there an effect of *CDKN2A/B* HemiDel in the propensity-score-matched cohort. These findings suggest that, in our cohort, the apparent prognostic effect of *CDKN2A/B* HemiDel in univariate analysis is mediated through other correlated factors such as preoperative and residual tumor volume. In fact, there was a trend in tumor volume being smaller in patients with retained status. This may indicate that tumors with a *CDKN2A/B* HemiDel are being more developed and further in the evolutionary trajectory of *IDH*-mutated lower-grade gliomas. Another possibility is that our study was underpowered to detect a modest independent effect of *CDKN2A/B* HemiDel, given the relatively small number of patients with this genetic alteration.

Further, our multivariable findings also prompt a re-evaluation of clinical factors such as WHO grade and age, which are currently incorporated into clinical decision making^18^^,^^33^ based on evidence that these factors influence survival.^19^^,^^20^ However, recent studies, including ours, suggest that their impact on survival may be less pronounced then previously assumed.^7^^,^^34–38^ This raises the question about the weight age and WHO grade (2 or 3) should be given in clinical practice in the management of *IDH*-mutated astrocytomas. Although our findings argue against a strong, independent prognostic effect of *CDKN2A/B* HemiDel, they indicate that *CDKN2A/B* status captures survival differences more consistently than WHO grade. This raises the possibility that *CDKN2A/B* status could contribute to refining diagnostic grading for astrocytomas.

In summary, our study extends current knowledge on the survival impact of *CDKN2A/B* by evaluating it alongside other clinically implemented prognostic markers. Furthermore, it provides some insights into the tumor biology of *CDKN2A/B* loss and how this deletion might be further incorporated into grading criteria for astrocytomas.

### Strength and Limitation Section

A key strength of this study is the use of multivariable analyses, which allowed us to account for several potential confounders and to evaluate whether the association between *CDKN2A/B* HemiDel and survival persisted after adjustment for clinical factors. This approach reduces the risk that our results are driven solely by imbalances in already established clinical factors. Another strength is the robustness of the *CDKN2A/B* assessment: copy-number status was evaluated twice and independently by 2 observers, which minimizes the risk of misclassification and increases confidence in the reliability of the molecular data.

Limitations are that we focused primarily on *CDKN2A/B* as a single molecular marker. Gliomas are biologically complex tumors with several genetic and epigenetic alterations, which could influence each other, and only accounting for one biomarker may not fully capture this heterogeneity. Another limitation is that despite a broad inclusion period, the overall cohort was small because of the rarity of the tumor. This limited sample size reduces statistical power. Together, these limitations underscore the need for larger multicenter studies incorporating comprehensive molecular profiling to validate and extend our findings.

## Conclusion

*CDKN2A/B* HemiDel was associated with worse survival in univariable analysis of astrocytomas, particularly in WHO grade 2. However, the independent contribution of *CDKN2A/B* HemiDel to prognosis remains uncertain, as no survival difference was seen after taking confounders into account. Larger multicenter studies are needed to clarify the prognostic role of *CDKN2A/B* HemiDel.

## Supplementary Material

vdag096_Supplementary_Data

## Data Availability

The data used for this article include confidential information, as in patient data, which is why they will not be published. Although parts of the data may be available upon reasonable request.
