# Supplementary material for: Hemizygous deletion of CDKN2A/B in IDH-mutated glioma: Prognostic impact when adjusting for clinical factors
Source: Neurooncol Adv. 2026 Apr 13;8(1):vdag096. doi: 10.1093/noajnl/vdag096 (PMC13152636; doi:10.1093/noajnl/vdag096)
Supplement: vdag096_Supplementary_Data [file vdag096_supplementary_data.docx]

**Supplementary material**

**
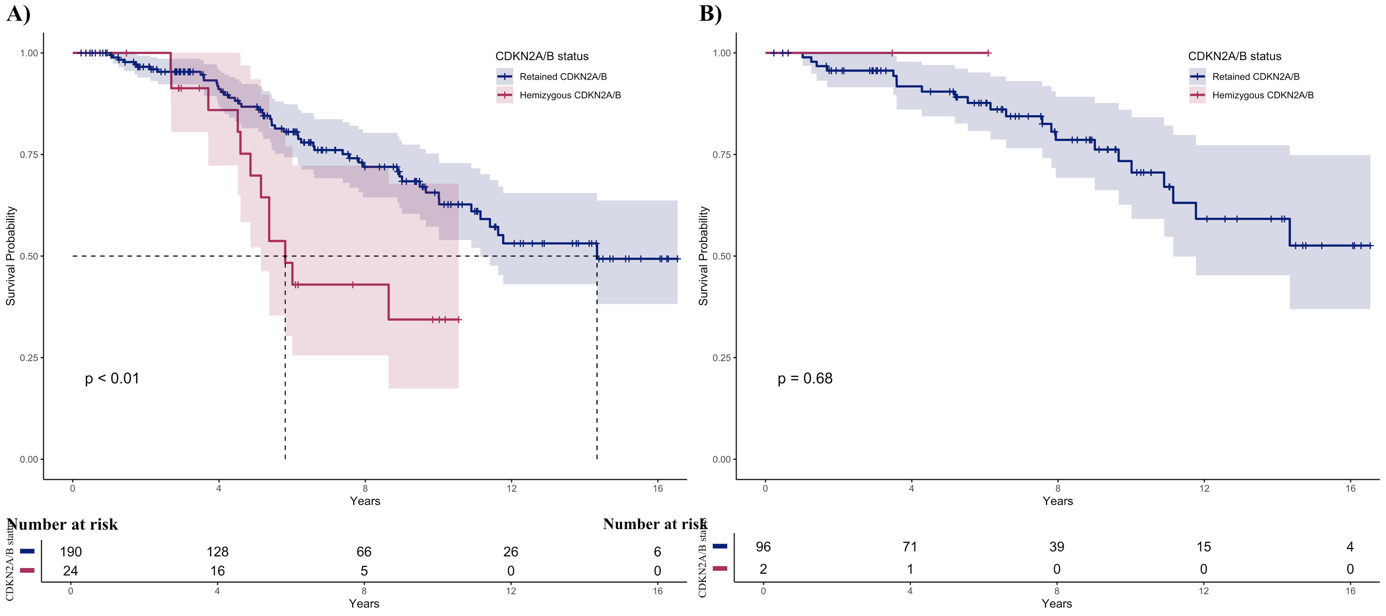
**

**Figure 1**: Survival analysis in IDH-mutated gliomas stratified by CDKN2A/B status. One patient with oligodendroglioma was excluded from the survival analysis due to loss to follow-up (A) Kaplan-Meier graph of the entire cohort consisting of both astrocytomas and oligodendrogliomas showed a significant survival effect of hemizygous deletion of CDKN2A/B (log-rank, p<0.01) (B) Subset analysis restricted to oligodendrogliomas did not show a survival difference depending on CDKN2A/B status (log-rank, p=0.68).

**Table 1**: Baseline characteristics of all IDH-mutated glioma patients included in this study.

|  | ***CDKN2A/B* status** | | |
| --- | --- | --- | --- |
|  | **HemiDel** | **Retained** | **p-value** |
| Total, n | 24 | 191 |  |
| Tumor subtype, n (%)  Astrocytoma  Oligodendroglioma | 22 (92)  2 (8) | 94 (49)  97 (51) | **< 0.001** |
| Male, n (%) | 16 (67) | 113 (59) | 0.51 |
| Age, Median (Q1, Q3) | 40 (33, 52) | 41 (33, 52) | 0.82 |
| Tumor grade, n (%)  Grade 2  Grade 3 | 10 (42)  14 (58) | 103 (54)  88 (46) | 0.28 |
| Symptoms at diagnosis, n (%)^a^  Seizures  Headache  Motor deficit  Language deficit  Visual deficit | 15 (63)  8 (33)  4 (17)  3 (13)  1 (4) | 146 (76)  29 (15)  18 (9)  18 (9)  4 (2) | 0.14  **0.04**  0.28  0.71  0.45 |
| Tumor location, n (%)  Frontal,  Temporal,  Other,  Bilateral, n (%) | 11 (46)  4 (17)  9 (38)  4 (17) | 116 (61)  39 (20)  36 (19)  20 (10) | 0.13  0.32 |
| Type of surgery, n (%)  Biopsy  Resection | 0 (0)  24 (100) | 7 (4)  184 (96) | 1.00 |
| Oncological treatment, n (%)  Combination treatment^b^  Chemotherapy or radiotherapy  No treatment | 16 (67)  6 (25)  2 (8) | 86 (45)  78 (41)  24 (13) | 0.20 |
| Tumor volume preoperatively, Median ml (Q1, Q3) | 73 (52, 92) | 49 (25, 86) | **0.03** |
| RANO, n (%)^c^  Class 1-2  Class 3  Class 4 | 6 (25)  8 (33)  9 (36) | 74 (39)  54 (28)  51 (27) | 0.35 |

^a^Several symptoms at time of diagnosis are possible, why >100 % is possible

^b^Radiotherapy with adjuvant chemotherapy or treatment according to the STUPP protocol

^c^Numbers do not reach 100% due to missing data.

**Table 2**: Standard mean difference (SMD) for the covariates before and after matching.

| **Group** | **All data**  **n = 76** | **Matched**  **N = 36** |
| --- | --- | --- |
| **Variable** | SMD | |
| Age | 0.224 | 0.020 |
| Grade 2 | -0.297 | 0.000 |
| Grade 3 | 0.297 | 0.000 |
| Preoperative segmentation volume | 0.275 | 0.049 |
| RANO class 1-2 | -0.526 | 0.000 |
| RANO class 3 | 0.206 | 0.000 |
| RANO class 4 | 0.297 | 0.000 |

**Table 3:** Baseline characteristics of the matched cohort.

|  | ***CDKN2A/B* status** | | |
| --- | --- | --- | --- |
|  | **HemiDel** | **Retained** | **p-value** |
| Total, n | 18 | 18 |  |
| Male, n (%) | 13 (72) | 10 (56) | 0.49 |
| Age, Median (Q1, Q3) | 35 (32, 49) | 38 (33, 42) | 0.77 |
| Tumor grade, n (%)  Grade 2  Grade 3 | 8(44)  10 (56) | 8 (45)  10 (56) | 1.00 |
| Symptoms at diagnosis, n (%)^a^  Seizures  Headache  Motor deficit  Language deficit  Visual deficit | 13 (72)  5 (28)  1 (6)  3 (17)  1 (6) | 17 (94)  3 (17)  3 (17)  1 (6)  0 (0) | 0.18  0.69  0.60  0.60  1.00 |
| Tumor location, n (%)  Frontal,  Temporal,  Other,  Bilateral, n (%) | 7 (39)  3 (17)  8 (45)  1 (6) | 9 (50)  5 (28)  4 (22)  2 (11) | 0.43  1.00 |
| Type of surgery, n (%)  Biopsy  Resection | 0 (0)  18 (100) | 0 (0)  18 (100) | 1.00 |
| Oncological treatment, n (%)  Combination treatment^b^  Chemotherapy or radiotherapy  Nothing | 13 (72)  4 (22)  1 (6) | 11 (61)  7 (39)  0 (0) | 1.00 |
| Tumor volume preoperatively, Median ml (Q1, Q3) | 71 (47, 101) | 52 (40, 101) | 0.60 |
| RANO, n (%)^c^  Class 1-2  Class 3  Class 4 | 5 (28)  8 (44)  5 (28) | 5 (28)  8 (44)  5 (28) | 1.00 |

^a^Several symptoms at time of diagnosis are possible, why >100 % is possible

^b^Radiotherapy with adjuvant chemotherapy or treatment according to the STUPP protocol

^c^Numbers do not reach 100% due to missing data.
